# Intrathecal immunoglobin synthesis and its role in patients with neurosyphilis

**DOI:** 10.3389/fpubh.2022.1008595

**Published:** 2022-11-07

**Authors:** Xiyue Huang, Shanshan Ying, Lan Luo, Lixin Li, Dongdong Li, Yi Xie

**Affiliations:** ^1^Division of Clinical Microbiology, Department of Laboratory Medicine, West China Hospital, Sichuan University, Chengdu, China; ^2^Department of Laboratory Medicine, West China Tianfu Hospital, Sichuan University, Chengdu, China

**Keywords:** intrathecal synthesis, neurosyphilis, immunoglobulins, cerebrospinal fluid, central nervous system

## Abstract

**Background:**

Intrathecal protein synthesis (ITS) occurs in various central nervous system disorders, but few quantitative studies have focused on ITS for neurosyphilis (NS) in southwestern China. We made a study to quantitatively assess the ITS in patients with NS and to investigate the association between ITS and the stages of NS.

**Methods:**

CSF–serum specimen pairs from 142 patients (66 NS and 76 non-NS/syphilis) were collected for routine CSF and serum tests. The NS group was divided into slight and severe subgroups according to the NS stages. Three formulas for the quantitative determination of the intrathecal synthesis were calculated to characterize the specimens, including the Ig index (Q_Ig_/Q_alb_), Ig extended index (Ig_EI), and intrathecally synthesized fraction (IgIF) using the hyperbolic function. The role of QTPPA/QIgG as an antibody index (AI = Q specific Ig/QIgG) was also explored.

**Results:**

Sero_TRUST titres (1:16, 1:1-1:256), sero_TPPA titres (1:163840, 1:1280-1:1310720), total protein (MTP), and CSF_Igs (*p* < 0.05) were found to be significantly elevated in the NS group. Intrathecal Ig synthesis can be identified using all three formulas in the NS group. The pattern of Ig intrathecal synthesis was IgIF-G (48.62%) > IgIF-A = IgIF-M (*p* < 0.05), with the dominant intrathecal fraction being IgG (median, 48.62%), which was also verified by Q_IgG_> Q_alb_> Q_IgM_ = Q_IgA_. In the slight NS group, the intrathecal fractions of IgM (>0 in 4 out of 20 cases) and IgG (>0 in 16 out of 20) were lower than the intrathecal fractions of IgM (>0 in 19 out of 35 cases) and IgG (>0 in 33 out of 38) in the severe group (*p* < 0.05). The area under the curve (AUC) of the CSF_TPPA antibody index was 0.867 (0.792, 0.922), with an optimal cutoff point of 0.81, providing a sensitivity of 88.91% and specificity of 84.62%.

**Conclusion:**

Although the intrathecal synthesis pattern is IgG dominant in patients with NS, brain-derived IgM and IgA can also be found. Moreover, intrathecal IgM and IgG were associated with a parenchymatous type of neurosyphilis. Syphilis-specific antibodies are a new potential tool for NS diagnosis.

## Introduction

Neurosyphilis (NS), syphilis of the central nervous system (CNS), has attracted a lot of attention in recent years (1, 2). NS has varied presentations and can be detected by laboratory tests. Diagnosis and treatment, as in previous eras, depend on clinical recognition. Guidelines from different countries, including Canada (3), Germany (4), the UK (5), and China, or organizations (6), such as the EU, highlight the role of cerebrospinal fluid (CSF) syphilis serology treponemal (TTs) and nontreponemal (nTTs) and CSF abnormalities. Due to the imperfect sensitivity and specificity of serology markers and the lack of benchmarks of CSF white-cell count (WBC) and protein level, the diagnosis of neurosyphilis is challenging from a clinical practical standpoint.

CSF_TPPA (Treponema *pallidum* particle agglutination assay) titers, CSF_CXCL13, and serological trust titers have the potential to diagnose or predict neurosyphilis in recent years (7–9). Additionally, nTTs and TTs might reflect CSF abnormalities before therapy, and the RPR response can succeed in predicting the normalization of CSF abnormalities after therapy (10). CSF syphilis serology is a breakthrough in the diagnosis of NS (11). The theoretical basis of CSF serology in NS is the role of humoral immunity in the inflammatory response and may be involved in CNS damage caused by *T. pallidum* (12), especially the role of ectopic germinal centers (EGCs). In other words, a brain-derived pathogen-specific antibody can be measured by the detection of intrathecal or local antibodies against *T. pallidum*. Due to impairment or selective function of the blood CSF barrier (BCB), it is difficult to discriminate the source of TTs and/or nTT antibodies, as immunoglobulins (Ig) in CSF might be brain-derived or blood-derived. Rieber and other researchers have developed formulas for assessing the intrathecal synthesis of immunoglobin quantitatively (13–16). The assessment of protein intrathecal synthesis (ITS) is an essential step in CSF analysis, especially pathogen-specific immunoglobin.

There are few quantitative studies focusing on ITSs in neurosyphilis in southwestern China, though *T. pallidum* expands and spreads globally (17). Here, we quantitatively assessed ITS in patients with NS and investigated the association between ITS and the stages of neurosyphilis. Additionally, the syphilis-specific response in the central nervous system was investigated. We hope that the intrathecal humoral response may be a future potential target for therapies or diagnoses that can help to prevent persistent infection and progression of the disease.

## Materials and methods

### Definitions

Neurosyphilis refers to syphilis patients with (1) CSF abnormalities: elevated CSF protein (>450 mg/L) and/or leukocyte count (>10 cells/μl) and (2) clinical symptoms or signs consistent with neurosyphilis without other known causes for these clinical abnormalities or a reactive CSF_TRUST. We divided the NS group into slight and severe subgroups according to the stages of neurosyphilis. Here, asymptomatic neurosyphilis, meningeal syphilis, meningovascular syphilis, and ocular syphilis were divided into the slight group, while general paresis and tabes dorsalis were classified into the severe group. Patients with syphilis without a confirmed diagnosis of neurosyphilis were defined as the non-neurosyphilis (N-NS) group.

#### Subjects and samples

In this study, we included 162 syphilis patients with CSF examinations between December 2019 and July 2021 at West China Hospital. The patients' basic characteristics were collected such as sex, age, syphilis stage, and HIV coinfection. The stages of syphilis were based on clinician diagnosis. Their residual CSF-serum specimen pairs were collected and stored at −80°C for subsequent analysis. CSF samples with macroscopic blood contamination or from duplicate patients were excluded. A total of 142 patients (66 patients with NS and 76 patients with N-NS) were ultimately included, whose information is illustrated in Table 1.

**Table 1:** Characteristic of the study population.

	**Neurosyphilis (*n* = 66)**	**Non-neurosyphilis/Syphilis (*n* = 76)**	***P***
Age (years), X ± SD	50.53 ± 12.37	51.27 ± 16.76	0.766
Male, n (%)	52 (78.79)	44 (57.89)	0.008
Syphilis stage (Latent-I-II-III)	12-0-1-53	63-2-1-10	0.000
HIV coinfection	2/66	7/76	0.132
Sera parameters
*Sero_IgA (g/L), X ± SD*	2.70 ± 0.97	2.96 ± 1.61	0.252
*Sero_IgG(g/L), X ± SD*	12.06 ± 2.74	12.08 ± 2.70	0.101
*Sero_IgM(g/L), X ± SD*	1.16 ± 0.48	1.33 ± 0.62	0.973
*Sero_TRD, median (range)*	1:16 (1:1,1:256)	1:2 (0, 1:128)	0.000
*Sero_TPPA titres, median (range)*	163,840 (1,280, 13,107,20)	5,120 (80, 327,680)	0.000
CSF parameters
*CSF_IgA(mg/L), median (Range)*	8.08 (0.20, 72.7)	3.13 (0.64, 133)	0.000
*CSF_IgG(g/L), median (Range)*	0.21 (0.017, 1.87)	0.035 (0.012 ,0.90)	0.000
*CSF_IgM(mg/L), median (Range)*	8.45 (0.18, 134)	0.42 (0.16, 92.8)	0.000
CSF_MTP(g/L), median (Range)	0.76 (0.26, 2.26)	0.41 (0.16, 4.9)	0.000
CSF_TPPA	1:20,480 (0, 1:655,360)	0 (0, 1:81,920)	0.000
CSF_TRD	1:2 (0, 1:128)	0 (0, 0)	0.000
CSF_WBC(10^6^/L)	10 (0, 430)	0 (0, 110)	0.000
CSF_glucose	3.62 ± 0.87	3.77 ± 0.99	0.328
CSF_Cl	125.67 ± 4.01	125.00 ± 4.29	0.345
Blood-CSF Barrier
*Sero_ALB(g/L), X ± SD*	39.17 ± 3.96	38.64 ± 4.82	0.503
*CSF_ALB(g/L), X ± SD*	0.36 ± 0.24	0.28 ± 0.29	0.097
Q_*alb*_ (*10^−3^)	9.57 ± 7.13	7.62 ± 8.69	0.165

### Tests

Some related clinical and routine laboratory parameters, including routine CSF results and serum results, were collected from the hospital information system (HIS) and laboratory information system (LIS). The syphilis serological tests, including TRUST (Rongsheng, China), TPPA (Fujirebio, Japan) tests, *Elecsys* Syphilis assays (Roche, German), and FTA-ABS (*Euroimmun*, German), were performed according to the manufacturer's instructions. CSF IgA and IgM were measured by Siemens Healthcare Diagnostics on a BN II platform (Siemens, German), while CSF IgG was measured by immunoturbidimetry (Beckman Coulter GmbH, America). Serum Igs were measured by immunoturbidimetry (Beckman Coulter GmbH, America). However, some values were missing, which may have been caused by a lower limit of detection in CSF (6.2 mg/L for albumin, 9.3 mg/L for IgG, 1.25 mg/L for IgA, and 0.13 mg/L for IgM).

### Formulas designed for the quantitative determination of intrathecal synthesis

The albumin quotient (Q_alb_) was used to evaluate the function of BCB. The IgG index (Q_Ig_/Q_alb_) is considered to be the most commonly used measure to evaluate the function of BCB and can be used in patients with normal BCB function (18). Since 1989, Ohman et al. (13, 16) improved the Ig index to the Ig extended index (Ig_EI = Q_Ig_/ ${\text{Q}}_{\text{alb}}^{\text{a}}$, constant *a*) based on the linear relationship between ln (Q_Ig_) and ln (Q_alb_). However, the groundwork of ITS laid by Reiber et al. is a hyperbolic curve between Q_Ig_ and Q_alb_ (15, 19–22). The ITS refers to the intrathecal fraction (IgIF) in % of total Ig in CSF (23). All three formulas can quantitatively determine the intrathecal synthesis, but in this study, we used an antibody index (AI = _QTPPA/QIgG_), providing indirect evidence for *T. pallidum* invasion in the CNS, to reflect the local pathogen-specific antibody response.

### Statistical analysis

The data were expressed as X ± SD or median (Range) according to its distribution. The unpaired *t*-test, Mann–Whitney *U* test, χ^2^ test or Fisher's exact test, linear regression, and logarithmic transformation were used in this study. The abovementioned statistical analyzes were performed using SPSS 26.0 for Windows or GraphPad Prism 8.30. Receiver operating characteristic (ROC) analysis was performed on MedCalc Statistical Software version 15.2.2 to determine the performance of the Treponema-specific antibody index, and the optimal cutoff was determined corresponding to the maximal Youden's index (sensitivity + specificity−100%). Missing values were not taken into the statistical calculations mentioned. A *p* < 0.05 was considered statistically significant.

### Ethics statements

This study was approved by (2020-920) the Ethics Committee of West China Hospital of Sichuan University.

## Results

### Characteristics of the study participants

In total, 66 patients with NS and 76 patients with N-NS were enrolled (Table 1). Nearly all patients with NS except one were reactive to CSF_TRUST (1:2,0-1:128), while all patients with N-NS were nonreactive with CSF_TRUST. There was no significant difference in age distribution between patients with N-NS and NS (*p* > 0.05). However, more male patients were seen in the NS group (*p* < 0.05). Higher sero_TRUST titres (1:16, 1:1-1:256) and sero_TPPA titres (1:163840,1:1280-1:1310720) were observed in the NS group (*p* < 0.001). No statistically significant differences were found in sero_Igs between the two groups (*p* > 0.05). Compared with CSF protein levels in the N-NS group, the total protein (MTP) and CSF_Igs were elevated in the NS group, but the ALB level was not elevated.

### Quantitative assessment of intrathecal Ig synthesis by different formulas

**Figure 1:**
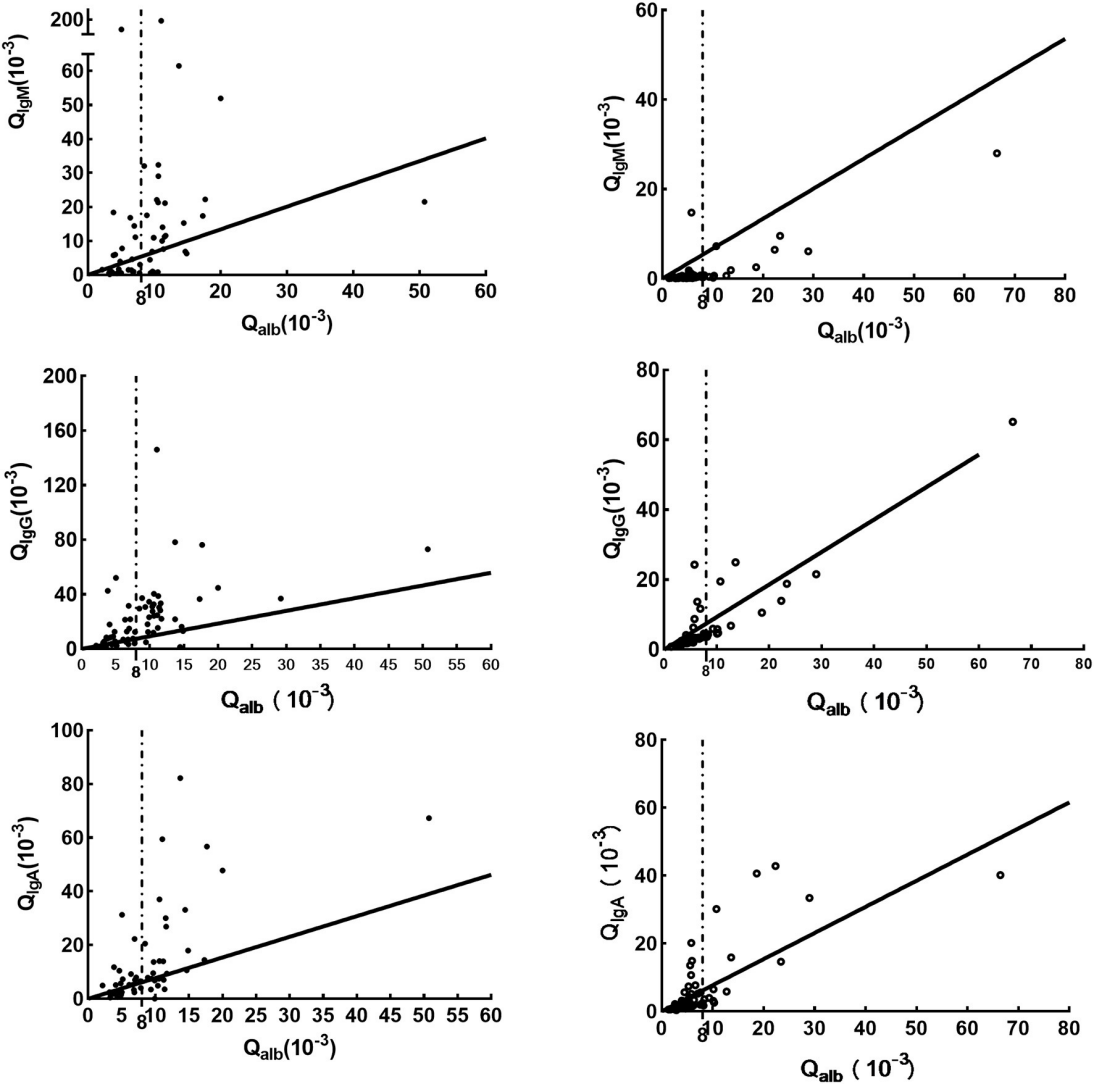
Ig quotient diagram with hyperbolic curves for the upper discrimination line (Q_Lim_) of the reference range for blood-derived Ig in CSF. The dark line is Qlim, the upper limit of the reference range for blood-derived Ig in CSF; the dashed line, indicates Qalb(10^−3^)>8; the solid point represents the cases in the NS group, while the hollow point indicates the cases in the N-NS group. Parameters for the calculation of the hyperbolic curves are obtained from a previous study (23).

Compared with data in the N-NS group, increased concentrations of the three CSF_Ig classes were seen in the NS group (Table 1). In the NS group, elevated Q_alb_ indicated BCB dysfunction (Table 1; *p* < 0.05). Intrathecal Igs can be found in the NS groups using all three formulas. However, more patients were found to have intrathecal Ig synthesis using the Ig index than those patients using the Ig extent index and IgIFs. In the N-NS group, the three intrathecal Ig syntheses were not so dissimilar among the three formulas. The molecular diffusion/CSF flow theory provides the sensitive identification of the intrathecal fraction of pathological protein (22, 24). When using the hyperbolic function, 79% of NS patients with brain-derived IgG and almost 50% of NS patients with brain-synthesized IgA and IgM. The pattern of Ig intrathecal synthesis was IgIF-G (48.62%) > IgIF-A = IgIF-M (*p* < 0.05), with the dominant intrathecal fraction being IgG (median, 48.62%) in the NS group, which was also verified by Q_IgG_ > Q_alb_ > Q_IgM_= Q_IgA_. The Ig quotient diagram with hyperbolic curves is presented in Figure 1.

### Association between brain injury and Ig synthesis

**Figure 2:**
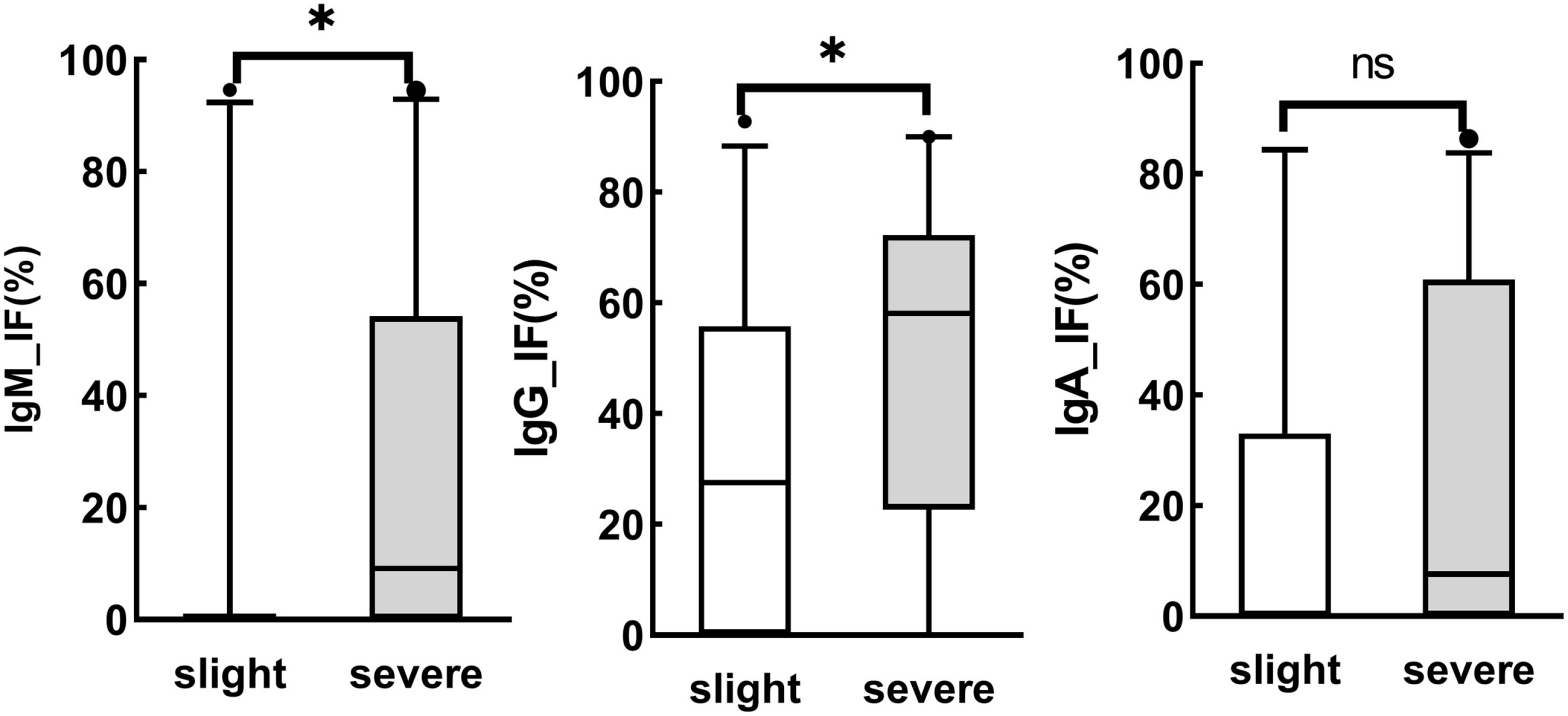
Association of brain injury and Ig synthesis. Asymptomatic neurosyphilis (*n* = 5), meningeal syphilis (*n* = 11), meningovascular syphilis (*n* = 6), and ocular syphilis (*n* = 3) were divided into the slight group, while general paresis (*n* = 38) and tabes dorsalis (*n* = 1) were classified into the severe group. ns, no significant. *P < 0.05.

Intrathecal IgM analysis is not considered a sign of the acute phase of a disease, as there is no Ig isotype switch in the brain (23). Moreover, the intrathecal IgM fraction is reported to be associated with a poor prognosis (25). As illustrated in Figure 2, the intrathecal fractions of IgM (4 out of 20 cases was >0) and IgG (16 out of 20 > 0) in the slight group were lower than the intrathecal fractions of IgM (19 out of 35 cases was >0) and IgG (33 out of 38 was >0) in the severe group (*p* < 0.05), while there was no statistically significant difference in the intrathecal fractions of IgA between the two groups. Moreover, intrathecal IgM and IgG were associated with general paresis and tabes dorsalis, late stages, or parenchymatous types of neurosyphilis.

### Role of TP antibody index in neurosyphilis

**Figure 3:**
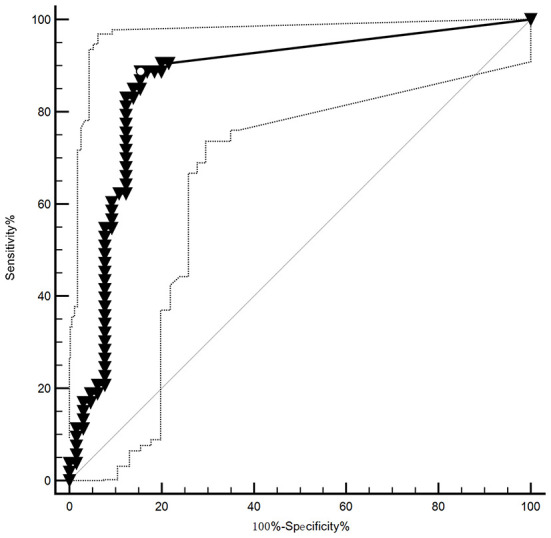
The ROC of the TP antibody index in neurosyphilis. The AUC of the CSF_TPPA index antibody index was AUC: 0.867 (0.792, 0.922), with its optimal cutoff points for the identification of NS being ≥0.81, providing a sensitivity of 88.91% and specificity of 84.62%.

The CSF_TPPA titer ≥ 1:320 or 1: 640 was specific for NS, but the CSF_TPPA index (CSF_TPPA/Sero_TPPA), which evaluates the intrathecal synthesis of treponemal antibody, has not yet been validated for the diagnosis. Here, the AUC of the CSF_TPPA index antibody index (Q_TPPA_/Q_IgG_) was 0.867 (0.792, 0.922), with an optimal cutoff of ≥0.81, a sensitivity of 88.91% and a specificity of 84.62% (Figure 3).

## Discussion

In neurosyphilis, although sero_TRUST titers and neurological symptoms were developed to predict neurosyphilis (26, 27), routine CSF analysis and CSF serology are still deemed the “gold” standard. Enrichment of B cells, especially EGCs, as well as differential expression of cytokines and chemokines, were observed in the CSF of patients with neurosyphilis in previous studies (12, 28–30), suggesting the important role of humoral or cellular immunity in neurosyphilis. In this study, we quantitatively assessed intrathecal immunoglobulin synthesis and explored its role in neurosyphilis.

Increased protein levels were significantly observed in the NS group (*p* < 0.05). Compared with CSF protein levels in the N-NS group, the MTP and CSF_Igs were elevated in the NS group (*p* < 0.05), but the ALB level was not elevated since all CSF albumin is blood-derived (*p* > 0.05), interfering with the elevation of immunoglobulins and other brain-derived proteins. Intrathecal synthesis of immunoglobulins occurs in many CNS disorders (23, 31), which can be demonstrated in two different ways: by isoelectric focusing electrophoresis (IFE) using CSF-Serum sample pairs and calculation of “synthesized” Igs using formulas. Unfortunately, IFE is often not available in most clinical laboratories.

In this study, three formulas were first used to assess the intrathecal immunoglobulin class in syphilis (Table 2). These three formulas showed that intrathecal fractions of CSF_IgM, CSF_IgG, and CSF_IgA were more common in the NS group than in the N-NS group (*p* < 0.05), suggesting the existence of brain-derived immunoglobulins. The discordant constituent ratio or pattern of ITS by different quantitative determinations is caused by different conditions of application of the three formulas in the NS group. The use of the Ig index assumes a linear relationship between the Q_Ig_ and the Q_alb_. Logarithmic transformations of Q_Ig_ and Q_alb_ did not result in a linear response in the NS group, indicating that Ig_EI may also not be suitable. Compared with the Ig index and Ig-EI, the hyperbolic function showed high sensitivity and a low rate of false-positive findings for central nervous inflammatory diseases (32). Here, the intrathecal synthesis is calculated as the intrathecal fraction (IgIF) in % of total Ig in CSF according to the hyperbolic function. A total of 43.36% of patients with NS showed intrathecal synthesis of IgM, 51.85% of patients with NS showed intrathecal synthesis of IgA and 79.03% of patients with NS showed intrathecal synthesis of CSF_IgG (Table 2). Hence, the pattern of intrathecal immunoglobulin in NS is IgG dominant, with occasional IgM and IgA intrathecal synthesis. Our data are consistent with a previous report that approximately 50% of patients with neurosyphilis show intrathecal synthesis of IgM (33), and in contrast with this previous report, the data show the absence of IgA synthesis in NS (23). IgA ITS does not necessarily discriminate neurosyphilis from other inflammatory CNS disorders. Moreover, intrathecal IgM and IgG were associated with general paresis and tabes dorsalis, late stages or parenchymatous types of neurosyphilis (Figure 2). There was a positive correlation among the CSF B cells, immunoglobulin indices, and CSF CXCL13 levels (12). CXCL13/CXCR5 mediated the aggregation of B cells, which directed the aberrant humoral immune responses *via* the formation of EGCs, suggesting neurological damage in neurosyphilis (12). Hence, the reasons for IgG and IgM associated with late stages or parenchymatous types may be aroused by the aberrant humoral immune responses. Although intrathecal IgG synthesis is the most frequent immune response detectable in 50% of the inflammations in the daily routine CSF analysis, the role of IgM ITS in the progressive paralysis of neurosyphilis should not be ignored.

**Table 2:** Quantitative assessment of Intrathecal immunoglobulin synthesis by different formulas.

	**Reference intervals**	**Neurosyphilis**		**Non-neurosyphilis/Syphilis**		***P***
		**(%) R/n**	**Median (Range)**	**(%) R/n**	**Median (Range)**	
**CSF/serum quotients: IgX index** **=** **Q**_**Ig**_**/ Q**_**alb**_						
IgG index (*10^**3**^**)**	≤0.62	87.09 (54/62)	1.95 (0.093, 13.21)	22.34 (15/67)	0.53 (0.37, 4.20)	0.000
IgM index	≤0.23	74.5 (41/55)	0.89 (0.043, 29.56)	13.33 (8/60)	0.059 (0.022, 2.55)	0.000
IgA Index	≤0.43	87.04 (47/54)	0.87 (0.0081, 6.22)	48.43 (31/64)	0.43 (0, 3.47)	0.000
**Extended indices (EI): IgX_EI** $=Q_{\text{Igx}}/Q_{\text{alb}}^{\text{a}}$ ,						
IgG-EI	≤1.24	87.09(54/62)	3.55 (0.15, 22.69)	17.9 (12/67)	0.99 (0.68, 7.77)	0.000
IgM-EI	≤15	81.82(45/55)	53.79 (3.69, 3457.48)	20 (12/60)	6.22 (1.37, 262.74)	0.000
IgA-EI	≤1.0	38.89 (21/54)	0.77 (0.02, 3.91)	6.25 (4/64)	0.45 (0.25, 1.79)	0.000
Intrathecally synthesized $IgX_{\text{i}}=(Q_{\text{Igx}}-a/b\sqrt{(Q_{\text{alb}}{}^{2}+b^{2}})+c)$*IgX (Ser). Constants: a/b, b^**2**^**,c ;IgIF** **=** IgXi/IgX(CSF)*100%						
IgIF-G	0	79.03 (49/62)	48.62 (0.92.80)	10.45 (7/67)	0 (0.75.90)	0.000
IgIF-M	0	43.36 (24/55)	0 (0,94.60)	1.67 (1/60)	0 (0, 43.82)	0.000
IgIF-A	0	51.85(28/54)	2.6(0,86.39)	18.75(12/64)	0 (0, 71.24)	0.000

The antibody index (AI), a quotient of CSF/serum albumin (Qalb) and immunoglobulin quotients (QIg), is useful to confirm a suspicion of infection in the CNS (31). Here, the CSF_TPPA index antibody index (QTPPA/QIgG), with a good AUC (0.867), suggested its potential diagnostic role in the NS. The cutoff (≥0.81) is different from the consensus that a cutoff ≥3 provides strong evidence of CNS infection (31). The CSF_TPPA antibody index has advantages, for all recent novel biomarkers, such as CXCL13 and other cytokines which are unspecific. Elevated CSF-TPHA titers/indices were associated with the signs of intrathecal Ig synthesis (34). Intrathecal anti-treponemal antibody production supports the diagnosis of active neurosyphilis, and with the use of the TPHA index, patients with abnormal CSF can be better classified in regard to their need for neurosyphilis therapy (35). However, careful interpretation of the AI result is still crucial due to the lack of a “gold standard” ideal cutoff, although measuring an AI for specific *T. pallidum* antibodies is a new potential tool for NS diagnosis (36). The high CSF treponemal-specific antibody response is a consequence of inflammatory pathology of the central nervous system (34). Further work on AI is needed.

In conclusion, the NS Ig ITS pattern was IgIF-G (48.62%) > IgIF-A = IgIF-M (*P* < 0.05), and intrathecal IgM and IgG were associated with general paresis and tabes dorsalis, late stages or parenchymatous types of neurosyphilis. The AI for specific *T. pallidum* antibodies is another new potential tool for NS diagnosis. However, there are several limitations in this study, including the small sample size, the possibility of misclassification of grouping error, and some missing data due to sample volume or detection limitation of assays. Also, we ignored the effect of HIV infection on intrathecal immunoglobulin synthesis and blood–cerebrospinal fluid barrier dysfunction due to only two cases of coinfection with HIV.

## Data availability statement

The raw data supporting the conclusions of this article will be made available by the authors, without undue reservation.

## Ethics statement

The Ethics Committee of West China Hospital of Sichuan University approved this study (2020-920). These samples were analyzed anonymously. For researchers, all personal or private information was blind. Written or oral informed consents can be exempted according to rules of the Ethics Committee of West China Hospital of Sichuan University. Written informed consent for participation was not required for this study in accordance with the national legislation and the institutional requirements.

## Author contributions

XYH: conceptualization, formal analysis, investigation, writing-original draft, and writing—review and editing. SSY: methodology, validation, and review. LXL and LL: investigation and review. YX: project administration. DDL: formal analysis, conceptualization, writing—review and editing, and supervision. All authors contributed to the article and approved the submitted version.

## Funding

The study was funded by Key R&D projects of the Sichuan Provincial Department of Science and Technology (2022YFS0309).

## Conflict of interest

The authors declare that the research was conducted in the absence of any commercial or financial relationships that could be construed as a potential conflict of interest.

## Publisher's note

All claims expressed in this article are solely those of the authors and do not necessarily represent those of their affiliated organizations, or those of the publisher, the editors and the reviewers. Any product that may be evaluated in this article, or claim that may be made by its manufacturer, is not guaranteed or endorsed by the publisher.

## References

[1] Ropper AH. Neurosyphilis. N Engl J Med. (2019) 381:1358–63. 10.1056/NEJMra1906228 31577877

[2] Ghanem KG Ram S Rice PA. The modern epidemic of syphilis. N Engl J Med. (2020) 382:845–54. 10.1056/NEJMra1901593 32101666

[3] Wong T Fonseca K Chernesky MA Garceau R Levett PN Serhir B. Canadian Public Health Laboratory Network laboratory guidelines for the diagnosis of neurosyphilis in Canada. Can J Infect Dis Med Microbiol. (2015) 26(Suppl. A):18A−22A. 10.1155/2015/167484 25798161 PMC4353983

[4] Klein M Angstwurm K Esser S Hahn K Maschke M Scheithauer S. German guidelines on the diagnosis and treatment of neurosyphilis. Neurol Res Pract. (2020) 2:33. 10.1186/s42466-020-00081-1 33225223 PMC7669305

[5] Kingston M French P Higgins S McQuillan O Sukthankar A Stott C. UK national guidelines on the management of syphilis 2015. Int J STD AIDS. (2016) 27:421–46. 10.1177/0956462415624059 26721608

[6] Janier M Hegyi V Dupin N Unemo M Tiplica GS Potočnik M. 2014 European guideline on the management of syphilis. J Eur Acad Dermatol Venereol. (2014) 28:1581–93. 10.1111/jdv.12734 25348878

[7] Shiva F Goldmeier D Lane P Ethiopia H Winston A. Cerebrospinal fluid TPPA titres in the diagnosis of neurosyphilis. Sex Transm Infect. (2020) 96:389–90. 10.1136/sextrans-2019-054198 31959701

[8] Li D Huang X Shi M Luo L Tao C. Diagnostic role of CXCL13 and CSF serology in patients with neurosyphilis. Sex Transm Infect. (2021) 97:485–9. 10.1136/sextrans-2020-054778 33436504

[9] Marra CM Maxwell CL Dunaway SB Sahi SK Tantalo LC. Cerebrospinal fluid treponema pallidum particle agglutination assay for neurosyphilis diagnosis. J Clin Microbiol. (2017) 55:1865–70. 10.1128/JCM.00310-17 28381602 PMC5442543

[10] Xiao Y Tong ML Lin LR Liu LL Gao K Chen MJ. Serological response predicts normalization of cerebrospinal fluid abnormalities at six months after treatment in HIV-negative neurosyphilis patients. Sci Rep. (2017) 7:9911. 10.1038/s41598-017-10387-x 28855625 PMC5577126

[11] Ghanem KG. Cerebrospinal fluid treponemal antibody titres: a breakthrough in the diagnosis of neurosyphilis. Sex Transm Infect. (2020) 96:391–2. 10.1136/sextrans-2020-054456 32033979

[12] Yu Q Cheng Y Wang Y Wang C Lu H Guan Z. Aberrant humoral immune responses in neurosyphilis: CXCL13/CXCR5 play a pivotal role for b-cell recruitment to the cerebrospinal fluid. J Infect Dis. (2017) 216:534–44. 10.1093/infdis/jix233 28931218

[13] Ohman S Ernerudh J Forsberg P von Schenck H Vrethem M. Improved formulae for the judgement of intrathecally produced IgA and IgM in the presence of blood CSF barrier damage. Ann Clin Biochem. (1993) 30 (Pt 5):454–62. 10.1177/000456329303000507 8250497

[14] Auer M Hegen H Zeileis A Deisenhammer F. Quantitation of intrathecal immunoglobulin synthesis-a new empirical formula. Eur J Neurol. (2016) 23:713–21. 10.1111/ene.12924 26806360

[15] Reiber H. Flow rate of cerebrospinal fluid (CSF)–a concept common to normal blood-CSF barrier function and to dysfunction in neurological diseases. J Neurol Sci. (1994) 122:189–203. 10.1016/0022-510X(94)90298-4 8021703

[16] Ohman S Forsberg P Nelson N Vrethem M. An improved formula for the judgement of intrathecally produced IgG in the presence of blood brain barrier damage. Clin Chim Acta. (1989) 181:265–72. 10.1016/0009-8981(89)90232-5 2758680

[17] Beale MA Marks M Cole MJ Lee MK Pitt R Ruis C. Global phylogeny of Treponema pallidum lineages reveals recent expansion and spread of contemporary syphilis. Nat Microbiol. (2021) 6:1549–60. 10.1038/s41564-021-01000-z 34819643 PMC8612932

[18] Lefvert AK Link H. IgG production within the central nervous system: a critical review of proposed formulae. Ann Neurol. (1985) 17:13–20. 10.1002/ana.410170105 3985580

[19] Reiber H Otto M Trendelenburg C Wormek A. Reporting cerebrospinal fluid data: knowledge base and interpretation software. Clin Chem Lab Med. (2001) 39:324–32. 10.1515/CCLM.2001.051 11388657

[20] Reiber H. Knowledge-base for interpretation of cerebrospinal fluid data patterns. Essentials in neurology and psychiatry. Arq Neuropsiquiatr. (2016) 74:501–12. 10.1590/0004-282x20160066 27332077

[21] Reiber H. Cerebrospinal fluid data compilation and knowledge-based interpretation of bacterial, viral, parasitic, oncological, chronic inflammatory and demyelinating diseases. Diagnostic patterns not to be missed in neurology and psychiatry. Arq Neuropsiquiatr. (2016) 74:337–50. 10.1590/0004-282X20160044 27097008

[22] Reiber H. Non-linear ventriculo-Lumbar protein gradients validate the diffusion-flow model for the blood-CSF barrier. Clin Chim Acta. (2021) 513:64–7. 10.1016/j.cca.2020.12.002 33316216

[23] Reiber H. Cerebrospinal fluid–physiology, analysis and interpretation of protein patterns for diagnosis of neurological diseases. Mult Scler. (1998) 4:99–107. 10.1177/135245859800400302 9762655

[24] Reiber H. Blood-cerebrospinal fluid (CSF) barrier dysfunction means reduced CSF flow not barrier leakage-conclusions from CSF protein data. Arq Neuropsiquiatr. (2021) 79:56–67. 10.1590/0004-282x-anp-2020-0094 33656113

[25] Stauch C Reiber H Rauchenzauner M Strasak A Pohl D Hanefeld F. Intrathecal IgM synthesis in pediatric MS is not a negative prognostic marker of disease progression: quantitative versus qualitative IgM analysis. Mult Scler. (2011) 17:327–34. 10.1177/1352458510388543 21123302

[26] Yan J Luo L Han J Yan D Zhang B Zhang Z. Comparing noninvasive predictors of neurosyphilis among syphilis patients with and without HIV co-infection based on the real-world diagnostic criteria: a single-center, retrospective cohort study in China. AIDS Res Hum Retroviruses. (2022) 38:406–14. 10.1089/aid.2021.0085 34314231

[27] Shiva F Short CE Goldmeier D Winston A. Predictive value of neurological symptoms in persons with suspected neurosyphilis. Sex Transm Infect. (2022) 98:228–9. 10.1136/sextrans-2021-055035 33875567

[28] Pastuszczak M Jakiela B Wielowieyska-Szybinska D Jaworek AK Zeman J Wojas-Pelc A. Elevated cerebrospinal fluid interleukin-17A and interferon-gamma levels in early asymptomatic neurosyphilis. Sex Transm Dis. (2013) 40:808–12. 10.1097/OLQ.0000000000000024 24275734

[29] Novikov YA Pervakova MY Lapin SV Titov AK Surkova EA Petrova NN. [Interleukins-23,−12p40 as markers of neural tissue damage in neurosyphilis.]. Klin Lab Diagn. (2019) 64:94–7. 10.18821/0869-2084-2018-64-2-94-97 30917250

[30] Yu XY Yan N Li ZH Chen W Hua YH Lu Y. [Expressions of CXCL8, CXCL10 and Th1 /Th2 cytokines in the serum and cerebrospinal fluid of patients with neurosyphilis]. Zhonghua Nan Ke Xue. (2020) 26:335–40. 10.13263/j.cnki.nja.2020.04.009 33351301

[31] Shamier MC Bogers S Yusuf E van Splunter M Ten Berge JCEM Titulaer M. The role of antibody indexes in clinical virology. Clin Microbiol Infect. (2021) 27:1207–11. 10.1016/j.cmi.2021.03.015 33813108

[32] Ohman S Ernerudh J Forsberg P Henriksson A von Schenck H Vrethem M. Comparison of seven formulae and isoelectrofocusing for determination of intrathecally produced IgG in neurological diseases. Ann Clin Biochem. (1992) 29(Pt 4):405–10. 10.1177/000456329202900406 1642446

[33] Ebinger M Grauer MT Uhr M. Intrathecal IgA synthesis in neurosyphilis. J Neurol Sci. (2005) 228:21–5. 10.1016/j.jns.2004.09.028 15607206

[34] Levchik N Ponomareva M Surganova V Zilberberg N Kungurov N. Criteria for the diagnosis of neurosyphilis in cerebrospinal fluid: relationships with intrathecal immunoglobulin synthesis and blood-cerebrospinal fluid barrier dysfunction. Sex Transm Dis. (2013) 40:917–22. 10.1097/OLQ.0000000000000049 24220351

[35] Tomberlin MG Holtom PD Owens JL Larsen RA. Evaluation of neurosyphilis in human immunodeficiency virus-infected individuals. Clin Infect Dis. (1994) 18:288–94. 10.1093/clinids/18.3.288 8011805

[36] Alberto C Deffert C Lambeng N Breville G Gayet-Ageron A Lalive P. Intrathecal synthesis index of specific anti-treponema IgG: a new tool for the diagnosis of neurosyphilis. Microbiol Spectr. (2022) 10:e0147721. 10.1128/spectrum.01477-21 35138118 PMC8826818

